# The top 100 cited articles in menstrual health among adolescent girls: a citation analysis

**DOI:** 10.1186/s12978-023-01656-2

**Published:** 2023-08-16

**Authors:** G. Alekhya, Dinesh Prasad Sahu, Priyamadhaba Behera

**Affiliations:** 1 Department of Community Medicine and Family Medicine, AIIMS Bhubaneswar, Bhubaneswar, Odisha India; 2 WHO NTEP, Bhubaneswar, Odisha India; 3 Department of Community Medicine and Family Medicine, AIIMS Bhubaneswar, 3rd Floor, Academic Block, Bhubaneswar, Odisha 751019 India

**Keywords:** Menstrual health, Adolescent girl, Citation analysis

## Abstract

**Background:**

Menstrual health is an important public health concern where it is still considered a taboo, and adolescent girls often lack knowledge about menstrual health, face limited access to sanitation facilities, and struggle with the affordability of sanitary materials. Every year numerous articles are published; however, only a few of them would be influential in the evolution of a particular field. The number of citations received by an article serves as a quality factor for the impact of the article in a particular field. Citation analysis analyses the relationship between citations received by articles. From the literature search, no citation analysis was conducted on menstrual health. Hence the objective of the study was to identify the articles which received hundred or more citations and also to identify the leading countries, journals, study designs, and departments conducting research on menstrual health.

**Methods:**

Citation analysis was done with search terms pertaining to adolescent and menstrual health using Google Scholar as a database in Publish or Perish software. The articles retrieved were exported to Microsoft Excel. Articles that received a hundred or more citations were screened for the type of article, department, and country where the study was conducted. A descriptive analysis of the hundred or more cited articles was done in Microsoft Excel.

**Results:**

A total of 982 articles pertaining to menstrual health among adolescent girls were retrieved. There were hundred articles with hundred and more citations pertaining to the menstrual health of adolescent girls. Cross-sectional study design, Obstetrics and Gynaecology department, India and USA countries, and PLOS ONE journal had the most citations in research on menstrual health among adolescent girls. The top ten articles were on menorrhagia, menstrual hygiene practices, Water, Sanitation and hygiene (WASH), stigma on menstruation, and education on menstrual health.

**Conclusion:**

The hundred cited articles on menstrual health among adolescent girls were mainly from high-income countries and were of more observational in nature than interventional. Thus, highlighting the need to strengthen experimental studies on the menstrual health of adolescent girls in Lower-middle-income countries.

## Plain Language Summary

Menstrual health is an important public health entity where menstrual health management remains poor among adolescent girls in lower-middle-income countries. From the literature, it has been observed that there is a lack of research pertaining to menstrual health. Research-related performances are evaluated through various objective measures; one of them is the number of citations received by the articles, which implicates the impact of the article in a particular field. Hence the objective of our study was to enlist the articles pertaining to menstrual health on adolescent health, which received hundred and more citations. The most common study design, department, country, and journal were determined from articles that received hundred and more citations. The study results showed that the Cross-sectional study design, Obstetrics and Gynaecology department, India and USA countries, and PLOS ONE journal conducted research with the highest number of articles having hundred and more citations. The top ten articles included research pertaining to domains such as menorrhagia, menstrual hygiene practices, Water, Sanitation, and hygiene (WASH), stigma on menstrual health, and education on menstrual health, which are imperative with respect to adolescent girls’ menstrual health. From the current citation analysis, it is evident that there is a need for strengthening research on menstrual health, which will generate evidence-based interventions and help policymakers implement necessary policies for adolescent health.

## Introduction

Menstrual health is defined as a state of complete physical, mental, and social well-being and not merely the absence of disease or infirmity in relation to the menstrual cycle [1]. Achieving menstrual health means that girls, women, and all other people who experience a menstrual cycle throughout their life course can access timely, accurate, age-appropriate information about menstruation and changes experienced throughout the life course, as well as related to hygiene practices and self-care [1]. Millions of girls and women worldwide experience period poverty, which is described as limited access to menstrual education, period products, or adequate water sanitation and hygiene (WASH) facilities [2]. In adolescent girls, the onset of menstruation is the start of a new phase, posing various issues such as harassment, stigma, and social exclusion. Menstrual health needs of adolescent girls go unmet due to a lack of access to basic services like toilets, poverty, gender inequality, and cultural taboos [3]. Also, menstrual health is imperative to improve global population health, achieve Sustainable Development Goals (SDGs), and attain gender equality and human rights by the year 2030 [4]. Although the current SDG framework does not explicitly mention menstrual health, various targets indirectly contribute to its attainment [5]. For example, SDG 3 focuses on good health and well-being, where providing knowledge about menstrual health and hygiene (MHH) leads to positive sexual and reproductive health outcomes. SDG 6 emphasizes clean water and sanitation, and ensuring facilities with safety and comfort for MHH will help in achieving this goal [5]. Various studies conducted worldwide found that menstrual hygiene practices remained poor in lower-middle-income countries (LMICs) [6]. Also, research pertaining to menstrual health in LMICs was more of participatory, qualitative, and descriptive methods, but a commensurate number of analytic studies have not been performed [6].

Over the years, the research-related performance of universities, as well as that of individual researchers, is increasingly evaluated through the use of objective measures. There is a growing awareness in research communities, government organizations, and funding bodies around the concept of evaluation metrics such as Informetrics, Bibliometrics, Scientometrics, Webometrics, and Altmetrics [7]. Bibliometrics are statistical analyses of books, articles, or other publications. Citation analysis is a commonly used bibliographic method. A citation implies a relationship between a part or the whole of the cited document and a part or the whole of the citing document [8]. Citation analysis is that area of bibliometrics that deals with the study of these relationships. It employs mathematical, statistical, comparison, abstraction, generalization, and logical methods to study citation patterns in scientific journals and papers [9]. Citation classics are highly cited publications identified by the Science Citation Index (SCI) and a publication cited more than 400 times; however, in some fields with fewer researchers, a hundred citations would qualify a work [10]. Although relying solely on citations may not be ideal for assessing the quality of scientific articles, a high number of citations is suggestive of utility by other researchers [7]. Various citation analysis have been conducted across different disciplines, such as Obstetrics and Gynaecology, critical care, orthopedics, dermatological, ophthalmology journals, citation classics in fertility and sterility, Uro-gynaecology, surgery, nephrology, in field of opioids, and in urethral construction [11–20]. However, no citation analysis specifically focused on menstrual health among adolescents was found. This gap in citation analysis represents an opportunity for exploring the existing literature on menstrual health and also contributes to the development of a comprehensive knowledge base on menstrual health and informs future research, policy, and practice in this important area.

Given the importance of the menstrual health of adolescents as a public health issue and the need for more analytic studies, the study aimed to identify and analyze top-cited articles on menstrual health among adolescents. By identifying the articles with more than a hundred citations, the authors aimed to identify key publications and seminal works that have contributed to the understanding of the menstrual health of adolescents, their challenges, and potential solutions over the decades. Also, the authors sought to identify the leading Journals publishing on menstrual health, the standard study designs employed, the departments involved, and the countries involved in research on menstrual health.

## Methods

We conducted a citation analysis in the field of menstrual health among adolescent girls to examine the patterns and frequency of citations of published articles.

### Bibliometric approach

The top 100 cited articles on menstrual health were identified as of March 2022 using a free, publicly accessible search software named Harzing’s Publish or Perish [21]. Publish, or Perish, is a software program that retrieves and analyzes the academic citation data provided by various data sources such as Google Scholar, Scopus, Web of Science, PubMed, etc. The software provides raw citations, analyzes these, and provides different metrics such as h-index, g-index, total numbers of citations, etc. The databases PubMed and Scopus cover only those articles published in Journals indexed with them, and it was reported that Web of Science provides citation counts from the journals listed in ISI Web of Science, thus underestimating the citation counts [22]. A study was conducted on Academic search engines and bibliographic databases (ASEBDS) comparing the sizes of twelve ASEBDS. Results showed that Google Scholar is currently the most comprehensive academic search engine that provides scholarly information with access to peer-reviewed academic journals and grey literature [23]. Publish or Perish software allows one to choose only one search engine or database for article retrieval. Hence, to test the study objectives, the authors have used Google Scholar as a search engine by considering the accessibility, feasibility, and provision of results with more accurate citation counts. We searched the articles using the search terms (adolescent OR adolescence OR puberty’ AND ‘Menstruation OR menstrual OR menstrual health OR menses’ AND ‘hygiene OR hygienically OR sanitation OR sanitary) by Google Scholar on Harzing’s Publish or Perish V6 software. The retrieval of articles was set at a maximum limit, and a total of 982 articles were retrieved. The results obtained were exported to Microsoft Excel from the software. Two researchers (AG and DPS) screened all articles independently. The articles had variables such as cites per year, authors, author count, title of the article, year of publication, journal name, and publisher; each article had URLs providing access to them. Manual extraction was done for variables such as country of origin, department conducting the study, and the type of study design by accessing the article URLs. For the variable country of origin, the authors have considered primarily the country where the study was conducted; if data was unavailable, the first author’s country was taken as the country of origin, and also countries we have classified the countries into a high-income, lower-income, lower middle income by using World Bank Classification. The variable department was considered from the affiliation of the primary author. The variable study design was considered if mentioned in the article’s title; if not, the researchers screened for materials and methods in abstract or full-text articles. In case of any discrepancy final consensus was arrived at after a discussion with the third researcher (PB). Once the data entry was complete, articles that received hundred or more citations were identified. The hundred or more cited articles were categorized as per the study design. All the hundred cited articles were in English language and were included for analysis.

### Statistical analysis

A descriptive analysis was performed for articles that received hundred or more citations using Microsoft Excel. Descriptive data were presented in percentage or proportion.

## Results

A total of 982 articles related to menstrual health among adolescent girls were retrieved using Publish or Perish software. The publication years ranged from 1910 to March 2022. Of the 982 articles, there were hundred articles that received hundred or more citations. Out of them, five articles received more than 400 citations and hence were considered citation classics [10]. The mean citation score was 212.9 ± 144.4, and the median of 170 (IQR: 138–246). Out of 982 articles, the minimum citation was one, and the maximum was 1270.

### Various domains with Rank one for articles that received 100 or more citations

Cross-sectional study is the most common study design, ranking first, with thirty-four articles having more than a hundred citations, followed by review articles and qualitative study. The Department of Obstetrics and Gynaecology ranked one with twenty-six articles, followed by the Department of Community Medicine and School of public health. The countries India and the USA are ranked first, with 24 articles, each having more than a hundred citations, followed by the United Kingdom and Ethiopia. PloS One is the leading journal that published six articles with more than a hundred citations, followed by BMJ Open and Obstetrics and Gynaecology (Table 1).

**Table 1:** Various domains which stand at Rank 1 for articles that received 100 or more citations

Variable	Rank 1	Total number of articles N = 100, n (%)
Study design	Cross-sectional study	34 (34)
Department	Department of Obstetrics and Gynaecology	26 (26)
Country	India & United States of America (USA)	24 (24)
Journal	PLOS ONE	6 (6)

### Characteristics of top ten articles with more than 100 citations

The top ten articles were in research pertaining to menorrhagia, menstrual hygiene, stigma pertaining to menstrual health, WASH, and education on menstrual health.

Citation classics: the topmost article received 1270 citations, assessed menstrual blood loss using a pictorial chart, and was published in BJOG: An International Journal of Obstetrics and Gynaecology journal in the year 1990; the department of Obstetrics and Gynaecology conducted the study. The study has measured the diagnostic accuracy of a pictorial chart in assessing menorrhagia. The article which ranked second had 639 citations, titled Menstrual Hygiene: how hygienic is an adolescent girl? This article was published in the Indian Journal of Community Medicine. The study was a cross-sectional study conducted in India by the Department of Community Medicine. The third article in the order received 514 citations which also determined menstrual blood loss conducted in Sweden. Fourth in order was a clinical report about the menstrual cycle as a vital sign which received 493 citations. The fifth citation classic was a Cochrane systematic review on antifibrinolytics for heavy menstrual bleeding (Table 2).

**Table 2:** Characteristics of top 10 articles having citations of more than 100 citations

Rank	Article title	Menstrual health domain	Citations	Publication year	Journal	Country
1	Assessment of menstrual blood loss using a pictorial chart [24]	Menorrhagia	1270	1990	BJOG: An International Journal of Obstetrics and gynecology	United Kingdom
2	Menstrual hygiene: how hygienic is the adolescent girl? [25]	Menstrual hygiene	639	2008	Indian Journal of Community Medicine	India
3	Determination of blood loss [26]	Menorrhagia	514	1964	Scandinavian Journal of Clinical and Laboratory Investigation	Sweden
4	Menstruation in girls and adolescents: using menstrual cycle as a vital sign [27]	Menstrual hygiene	493	2006	Pediatrics	USA
5	Antifibrinolytic for heavy menstrual bleeding [28]	Menorrhagia	411	2000	Cochrane database of systematic reviews	New Zealand
6	Treatment of menorrhagia during menstruation: randomized controlled trial of mefenamic acid and tranexamic acid [29]	Menorrhagia	384	1996	BMJ open	Ireland
7	Menstrual hygiene: Knowledge and practice among adolescent schoolgirls of Nagpur district [30]	Menstrual hygiene	379	2011	Journal of the clinic and diagnostic research	India
8	Where the education system and women’s bodies collide: The health impact of girls’ experiences of menstruation in Tanzania [31]	Education on menstrual health	357	2010	Journal of adolescence	Tanzania
9	Menstrual practices and reproductive problems: a. study of adolescent girls in Rajasthan [32]	WASH	351	2015	Journal of Health Management	India
10	The menstrual mark: menstruation as social stigma [33]	Social stigma on menstruation	341	2013	Sex roles	United Kingdom

**Fig. 1:**
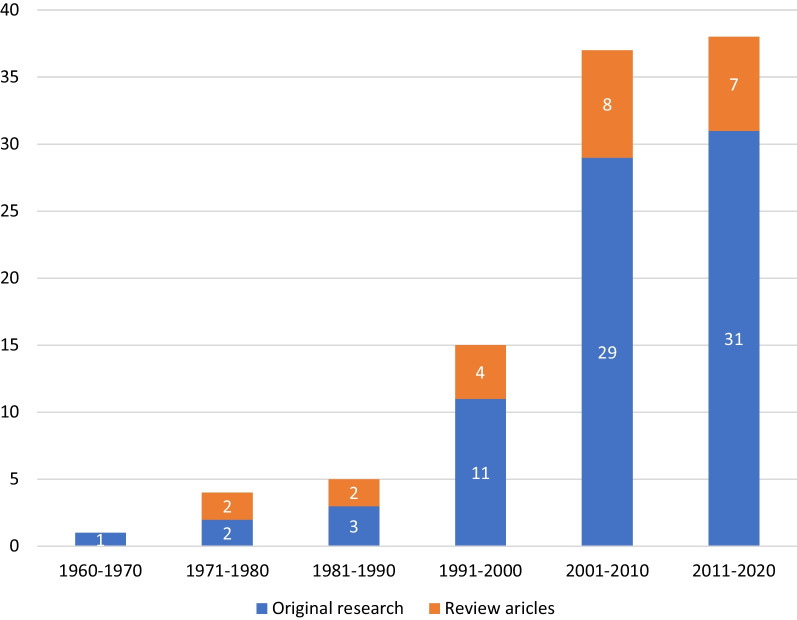
Top hundred cited articles in research on menstrual health by the decade of publication

**Fig. 2:**
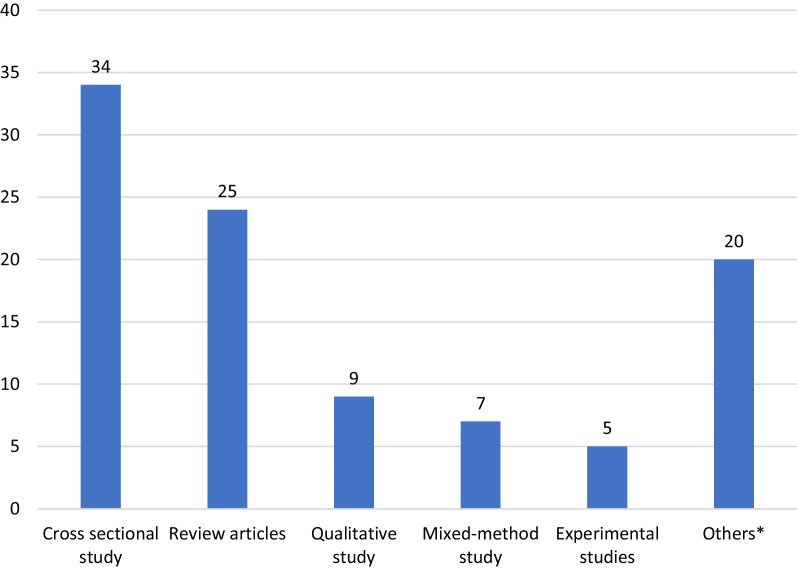
Top 100 cited articles categorized based on study design. *Others include cohort study (2), case–control study (1), commentaries, and editorials

Trend analysis of the top hundred cited articles showed that original and review articles shared equal proportions in earlier decades, and an increase in trend was noted in the decade 2011–2020; the proportion of original research increased relatively when compared to review articles in the coming decades (Fig. 1). Most of the top hundred cited articles were cross-sectional studies [34], followed by reviews [25]. There were five experimental studies in the top hundred cited articles; one was quasi-experimental, and the other four were randomized controlled trials (Fig. 2). Out of the top hundred articles, fifty-three were in higher income countries, of which the majority were from the USA [24], and the rest, forty-seven were from LMICs, with India being the major contributor [24]. Out of five experimental studies, four were from higher-income countries, and one was from LMICs (Nepal).

## Discussion

The authors conducted a citation analysis to identify articles with a hundred or more citations in the field of menstrual health among adolescent girls published from the year 1910 till March 2022. Several disciplines have conducted citation analysis in the medical field to understand the scientific progress of the field [11–20]. The current citation analysis provides an overview of how research pertaining to menstrual health among adolescents has duly evolved over time. Various databases, such as the Science citation index, Scopus, and Web of Science, have been used in previous studies to retrieve relevant articles [11–14, 18–20]. In the current study, the authors extensively reviewed the literature extensively and identified that Google Scholar, as a search engine provides comprehensive scholarly information that provides access to peer-reviewed journal publications and grey literature. Most of the top hundred cited articles were cross-sectional studies that focused on menstrual hygiene practices among adolescent girls, indicating that research from various countries over the decade has emphasized this aspect. However, there were only five interventional studies that assessed the effectiveness of interventions, such as providing sanitary pads to reduce school absenteeism and using drugs to reduce menstrual bleeding [29, 34]. These interventional studies were published in high-impact journals such as BMJ Open, highlighting the need for more rigorous methodological interventional studies to improve menstrual health outcomes among adolescent girls.

The Department of Obstetrics and Gynaecology was the top department conducting research on menstrual health among adolescent girls; this finding was expected as gynecologists often specialize in treating menstrual disorders and providing health education on menstrual hygiene. The top journals publishing literature on menstrual health include PLOS ONE, BMJ Open, and the Indian Journal of Community Medicine. However, few articles from these journals were not open-access, suggesting that authors tend to cite articles from journals with high-impact factors even if full-text access is not available [24, 26].

From the current citation analysis, several important findings have emerged. The top ten articles which received more than hundred citations, research were in domains such as menorrhagia, menstrual hygiene practices, stigma on menstruation, WASH (Water, Sanitation, and Hygiene) interventions, and education related to menstrual health. These findings are concurrent with the societal needs regarding menstrual health. Within the menorrhagia domain, the analysis observed that various aspects, including the determination of menstrual blood loss and the management of menorrhagia through different drugs were prominently featured. This indicates that these citations align more closely with the specific research needs in this area. Among the top ten of the hundred cited articles, the publication years ranged from 1964 to 2008. While citations generally accumulate over time, it is worth noting that the most cited article among the top ten was published in 1990 and focused on assessing menstrual blood loss in the context of menorrhagia. However, the second most cited article, published in the year 2008, introduced a new domain, which is menstrual hygiene. This suggests a shift in focus from menorrhagia to menstrual hygiene in subsequent years. Therefore, it can be inferred that factors beyond time, such as the relevance of research to current needs, play a significant role in citations. It is interesting to highlight that although the journal PLOS ONE ranked first among the hundred cited articles, there were no articles found from the journal in the top ten.

The impact of articles in later years compared to previous years can be observed through citation classics. In the context of citation classics, it has been found that out of five citation classics, three were published in the year 2000. This demonstrates that articles from later years had a significant impact when compared to previous years. This trend of high impact in later years can be attributed to an increase in original research in the later decades. Similar findings were also noted in a study with respect to low citation numbers and publication period, where the reason stated that this could be due to inefficient research during the timeline [35]. Also, among the five citation classics, two articles were not open access. These two articles focused on the assessment of menstrual loss and were among the most highly cited articles. The lack of open access availability for these articles can potentially limit access to scientific literature.

As per the literature, there was a research gap found in high-income and LMICs, and studies in LMICs were more off descriptive studies than analytical; interestingly, our study results also provided the same evidence. The majority of the top hundred cited were from higher-income countries such as the USA, United Kingdom, and Canada. Although India being a LMIC country, ranked top along with the USA, the studies were more cross-sectional studies, and none of them were interventional studies. Out of five, four interventional studies were from high-income countries. Thus, there is a need to prioritize research in LMICs to improve menstrual health outcomes among adolescent girls. The, current citation analysis enlightens on the amalgam of research conducted in menstrual health, which helped in analyzing the most common countries and studies deployed in the research as well as lacunae that can be addressed in the future.

## Strengths and limitations

The current citation analysis addressed an important public health entity, menstrual hygiene; as per the literature search, there were no citation analyses done in this field; this is the first citation analysis that provides an overview of various countries and departments conducting the research pertaining to menstrual health. The analysis included articles without limiting to a year of publication, and all types of articles were included, which included narrative reviews, cross-sectional studies, interventional studies, and mixed-method studies.

The study has certain limitations that should be acknowledged. It is recommended to use more than one search engine or database [36]. However, we were constrained to use only Google Scholar due to the limitations of the Publish or Perish software, which allows the use of only one search engine or database. It is essential to note that databases such as PubMed, Scopus, and Web of Science have distinct advantages over Google Scholar when it comes to ensuring the quality and reliability of the included articles. During future citation analysis, it is better to incorporate multiple databases, to ensure a comprehensive and robust analysis of the literature. Additionally, citation analysis has its own limitations. The effect accessibility of articles can influence the citation counts. Moreover, the concept of the Matthew effect introduces bias, as—high-impact journals and articles by eminent scientists tend to receive more citations when compared to unknown researchers or articles published in low-impact journals [37]. It is important to note that high citation counts are not always a measure of quality and can even be attributed to negative examples. Further ethnic biases may exist due to the similarity of study participants. To gain a deeper understanding and perform a more comprehensive analysis of the articles, further bibliometric analysis can be conducted.

## Conclusion

In the present citation analysis, descriptive analysis was conducted on articles with a hundred or more citations in the field of menstrual health. Among the hundred and more cited articles, India and USA countries, PLOS ONE journal, Obstetrics and Gynecology department, Cross-sectional study design ranked first pertaining to menstrual health among adolescent girls. The top ten articles encompassed various areas of menstrual health, such as menorrhagia, menstrual hygiene practices, the stigma associated with menstruation, WASH, and education on menstrual health. The current citation analysis provides valuable insights into the origin and trends of research in the field of menstrual health. The analysis also highlighted that menstrual hygiene practices remain poor in lower-income countries. It emphasizes the need for interventional studies to generate evidence and address the challenges regarding menstrual health in these countries. Strengthening research efforts in lower-income countries and conducting more interventional studies is crucial to improve menstrual hygiene practices and overall menstrual health.

## Data Availability

All data generated or analyzed during the current study are available from the corresponding author upon reasonable request.

## Appendix: Top 100 cited articles on menstrual health among adolescent girls

**Table:**

Rank	Citations	Article
1	**1270**	Higham JM, O'brien PM, Shaw R. Assessment of menstrual blood loss using a pictorial chart. BJOG: An International Journal of Obstetrics & Gynaecology. 1990 Aug;97(8):734–9
2	**639**	Dasgupta A, Sarkar M. Menstrual hygiene: how hygienic is the adolescent girl?. Indian journal of community medicine: official publication of Indian Association of Preventive & Social Medicine. 2008 Apr;33(2):77
3	**514**	Hallberg L, Nilsson L. Determination of menstrual blood loss. Scandinavian Journal of Clinical and Laboratory Investigation. 1964 Jan 1;16(2):244–8
4	**493**	Diaz AM, Laufer MR, Breech LL. Menstruation in girls and adolescents: using the menstrual cycle as a vital sign. Pediatrics. 2006 Nov 1;118(5):2245–50
5	**411**	Bryant‐Smith AC, Lethaby A, Farquhar C, Hickey M, Cochrane Gynaecology and Fertility Group. Antifibrinolytics for heavy menstrual bleeding. Cochrane database of systematic reviews. 1996 Sep 1;2018(6)
6	**384**	Bonnar J, Sheppard BL. Treatment of menorrhagia during menstruation: randomised controlled trial of ethamsylate, mefenamic acid, and tranexamic acid. Bmj. 1996 Sep 7;313(7057):579–82
7	**379**	Thakre SB, Thakre SS, Reddy M, Rathi N, Pathak K, Ughade S. Menstrual hygiene: knowledge and practice among adolescent school girls of Saoner, Nagpur district. J Clin Diagn Res. 2011 Oct 1;5(5):1027–33
8	**357**	Sommer M. Where the education system and women’s bodies collide: the social and health impact of girls’ experiences of menstruation and schooling in Tanzania. Journal of adolescence. 2010 Aug 1;33(4):521–9
9	**351**	Khanna A, Goyal RS, Bhawsar R. Menstrual practices and reproductive problems: a study of adolescent girls in Rajasthan. Journal of health management. 2005 Apr;7(1):91–107
10	**341**	Johnston-Robledo, I., Chrisler, J.C. The Menstrual Mark: Menstruation as Social Stigma. *Sex Roles* 68, 9–18 (2013). 10.1007/s11199-011-0052-z
11	**339**	Janssen CA, Scholten PC, Heintz AP. A simple visual assessment technique to discriminate between menorrhagia and normal menstrual blood loss. Obstetrics & Gynecology. 1995 Jun 1;85(6):977–82
12	**332**	Sumpter C, Torondel B. A systematic review of the health and social effects of menstrual hygiene management. PloS one. 2013 Apr 26;8(4):e62004
13	**326**	Brinton LA, Hamman RF, Huggins GR, Lehman HF, Levine RS, Mailin K, Fraumeni Jr JF. Sexual and reproductive risk factors for invasive squamous cell cervical cancer. Journal of the National Cancer Institute. 1987 Jul 1;79(1):23–30
14	**313**	McMahon SA, Winch PJ, Caruso BA, Obure AF, Ogutu EA, Ochari IA, Rheingans RD. ‘The girl with her period is the one to hang her head’ Reflections on menstrual management among schoolgirls in rural Kenya. BMC international health and human rights. 2011 Dec;11:1–0
15	**301**	Mahon T, Fernandes M. Menstrual hygiene in South Asia: a neglected issue for WASH (water, sanitation and hygiene) programmes. Gender & Development. 2010 Mar 1;18(1):99–113
16	**301**	Bernstein L, Ross RK, Lobo RA, Hanisch R, Krailo MD, Henderson BE. The effects of moderate physical activity on menstrual cycle patterns in adolescence: implications for breast cancer prevention. British journal of cancer. 1987 Jun;55(6):681–5
17	**281**	Lukes AS, Moore KA, Muse KN, Gersten JK, Hecht BR, Edlund M, Richter HE, Eder SE, Attia GR, Patrick DL, Rubin A. Tranexamic acid treatment for heavy menstrual bleeding: a randomized controlled trial. Obstetrics & Gynecology. 2010 Oct 1;116(4):865–75
18	**276**	Chimbira TH, Anderson AB, Turnbull AC. Relation between measured menstrual blood loss and patient's subjective assessment of loss, duration of bleeding, number of sanitary towels used, uterine weight and endometrial surface area. BJOG: An International Journal of Obstetrics & Gynaecology. 1980 Jul;87(7):603–9
19	**275**	El-Gilany AH, Badawi K, El-Fedawy S. Menstrual hygiene among adolescent schoolgirls in Mansoura, Egypt. Reproductive health matters. 2005 Jan 1;13(26):147–52
20	**270**	Van Eijk AM, Sivakami M, Thakkar MB, Bauman A, Laserson KF, Coates S, Phillips-Howard PA. Menstrual hygiene management among adolescent girls in India: a systematic review and meta-analysis. BMJ open. 2016 Mar 1;6(3):e010290
21	**270**	Jasper C, Le TT, Bartram J. Water and sanitation in schools: a systematic review of the health and educational outcomes. International journal of environmental research and public health. 2012 Aug;9(8):2772–87
22	**266**	Oster E, Thornton R. Menstruation, sanitary products, and school attendance: evidence from a randomized evaluation. American Economic Journal: Applied Economics. 2011 Jan 1;3(1):91–100
23	**253**	Thomas SL, Ellertson C. Nuisance or natural and healthy: should monthly menstruation be optional for women?. The Lancet. 2000 Mar 11;355(9207):922–4
24	**249**	Mason L, Nyothach E, Alexander K, Odhiambo FO, Eleveld A, Vulule J, Rheingans R, Laserson KF, Mohammed A, Phillips-Howard PA. ‘We keep it secret so no one should know’–A qualitative study to explore young schoolgirls attitudes and experiences with menstruation in rural Western Kenya. PloS one. 2013 Nov 14;8(11):e79132
25	**247**	Zegeye DT, Megabiaw B, Mulu A. Age at menarche and the menstrual pattern of secondary school adolescents in northwest Ethiopia. BMC women's health. 2009 Dec;9(1):1–8
26	**246**	Chandra-Mouli V, Patel SV. Mapping the knowledge and understanding of menarche, menstrual hygiene and menstrual health among adolescent girls in low-and middle-income countries. The Palgrave handbook of critical menstruation studies. 2020:609–36
27	**240**	Titus-Ernstoff L, Perez K, Cramer DW, Harlow BL, Baron JA, Greenberg ER. Menstrual and reproductive factors in relation to ovarian cancer risk. British journal of cancer. 2001 Mar;84(5):714–21
28	**239**	Tegegne TK, Sisay MM. Menstrual hygiene management and school absenteeism among female adolescent students in Northeast Ethiopia. BMC public health. 2014 Dec;14(1):1–4
29	**238**	Garg S, Sharma N, Sahay R. Socio-cultural aspects of menstruation in an urban slum in Delhi, India. Reproductive health matters. 2001 Jan 1;9(17):16–25
30	**236**	Sommer M, Sahin M. Overcoming the taboo: advancing the global agenda for menstrual hygiene management for schoolgirls. American journal of public health. 2013 Sep;103(9):1556–9
31	**230**	Dhingra R, Kumar A, Kour M. Knowledge and practices related to menstruation among tribal (Gujjar) adolescent girls. Studies on Ethno-Medicine. 2009 Jan 1;3(1):43–8
32	**230**	Adinma ED, Adinma JI. Perceptions and practices on menstruation amongst Nigerian secondary school girls. African journal of reproductive health. 2008 Apr 1;12(1):74–83
33	**229**	Das P, Baker KK, Dutta A, Swain T, Sahoo S, Das BS, Panda B, Nayak A, Bara M, Bilung B, Mishra PR. Menstrual hygiene practices, WASH access and the risk of urogenital infection in women from Odisha, India. PloS one. 2015 Jun 30;10(6):e0130777
34	**222**	Sahoo KC, Hulland KR, Caruso BA, Swain R, Freeman MC, Panigrahi P, Dreibelbis R. Sanitation-related psychosocial stress: a grounded theory study of women across the life-course in Odisha, India. Social science & medicine. 2015 Aug 1;139:80–9
35	**221**	Sommer M, Caruso BA, Sahin M, Calderon T, Cavill S, Mahon T, Phillips-Howard PA. A time for global action: addressing girls’ menstrual hygiene management needs in schools. PLoS medicine. 2016 Feb 23;13(2):e1001962
36	**217**	Lawan UM, Nafisa WY, Musa AB. Menstruation and menstrual hygiene amongst adolescent school girls in Kano, Northwestern Nigeria. African journal of reproductive health. 2010 Sep 1;14(3):201–7
37	**209**	Sommer M, Hirsch JS, Nathanson C, Parker RG. Comfortably, safely, and without shame: defining menstrual hygiene management as a public health issue. American journal of public health. 2015 Jul;105(7):1302–11
38	**204**	Cole SK, Billewicz WZ, Thomson AM. Sources of variation in menstrual blood loss. BJOG: An International Journal of Obstetrics & Gynaecology. 1971 Oct;78(10):933–9
39	**199**	Hennegan J, Montgomery P. Do menstrual hygiene management interventions improve education and psychosocial outcomes for women and girls in low and middle income countries? A systematic review. PloS one. 2016 Feb 10;11(2):e0146985
40	**197**	Adhikari P, Kadel B, Dhungel SI, Mandal A. Knowledge and practice regarding menstrual hygiene in rural adolescent girls of Nepal. Kathmandu University medical journal (KUMJ). 2007 Jul 1;5(3):382–6
41	**197**	Reid PC, Coker A, Coltart R. Assessment of menstrual blood loss using a pictorial chart: a validation study. BJOG: An International Journal of Obstetrics & Gynaecology. 2000 Mar;107(3):320–2
42	**195**	Ali TS, Rizvi SN. Menstrual knowledge and practices of female adolescents in urban Karachi, Pakistan. Journal of adolescence. 2010 Aug 1;33(4):531–41
43	**191**	Mudey AB, Kesharwani N, Mudey GA, Goyal RC. A cross-sectional study on awareness regarding safe and hygienic practices amongst school going adolescent girls in rural area of Wardha District, India. Global journal of health science. 2010 Oct 1;2(2):225
44	**187**	Kumar A, Srivastava K. Cultural and social practices regarding menstruation among adolescent girls. Social work in public health. 2011 Sep 15;26(6):594–604
45	**182**	Montgomery P, Ryus CR, Dolan CS, Dopson S, Scott LM. Sanitary pad interventions for girls’ education in Ghana: a pilot study. PloS one. 2012 Oct 31;7(10):e48274
46	**182**	Miller L, Notter KM. Menstrual reduction with extended use of combination oral contraceptive pills: randomized controlled trial. Obstetrics & Gynecology. 2001 Nov 1;98(5):771–8
46	**176**	Wyatt KM, Dimmock PW, Walker TJ, O’Brien PS. Determination of total menstrual blood loss. Fertility and sterility. 2001 Jul 1;76(1):125–31
47	**176**	Kissling EA. Bleeding out loud: Communication about menstruation. Feminism & Psychology. 1996 Nov;6(4):481–504
48	**176**	Shanbhag D, Shilpa R, D'Souza N, Josephine P, Singh J, Goud BR. Perceptions regarding menstruation and practices during menstrual cycles among high school going adolescent girls in resource limited settings around Bangalore city, Karnataka, India. International Journal of Collaborative Research on Internal Medicine & Public Health. 2012 Jul 1;4(7):1353
49	**172**	Garg R, Goyal S, Gupta S. India moves towards menstrual hygiene: subsidized sanitary napkins for rural adolescent girls—issues and challenges. Maternal and child health journal. 2012 May;16:767–74
50	171	Jensen JT, Parke S, Mellinger U, Machlitt A, Fraser IS. Effective treatment of heavy menstrual bleeding with estradiol valerate and dienogest: a randomized controlled trial. Obstetrics & Gynecology. 2011 Apr 1;117(4):777–87
51	**169**	Devi KD, Ramaiah PV. A study on menstrual hygiene among rural adolescent girls. Indian journal of medical sciences. 1994 Jun;48(6):139–43
52	**164**	Walker A. The Menstrual cycle.2008 Mar 7 [cited 2023 Jun 21]; https://www.taylorfrancis.com/books/mono/10.4324/9780203135051/menstrual-cycle-anne-walker
53	**164**	Treloar SA, Bell TA, Nagle CM, Purdie DM, Green AC. Early menstrual characteristics associated with subsequent diagnosis of endometriosis. American journal of obstetrics and gynecology. 2010 Jun 1;202(6):534-e1
54	**162**	Jewitt S, Ryley H. It’s a girl thing: menstruation, school attendance, spatial mobility and wider gender inequalities in Kenya. Geoforum. 2014 Sep 1;56:137–47
55	**161**	Demba E, Morison L, Van der Loeff MS, Awasana AA, Gooding E, Bailey R, Mayaud P, West B. Bacterial vaginosis, vaginal flora patterns and vaginal hygiene practices in patients presenting with vaginal discharge syndrome in The Gambia, West Africa. BMC infectious diseases. 2005 Dec;5(1):1–2
56	**161**	Agarwal A, Venkat A. Questionnaire study on menstrual disorders in adolescent girls in Singapore. Journal of pediatric and adolescent gynecology. 2009 Dec 1;22(6):365–71
57	**158**	Costos D, Ackerman R, Paradis L. Recollections of menarche: communication between mothers and daughters regarding menstruation. Sex roles. 2002 Jan;46:49–59
58	**156**	Kaur R, Kaur K, Kaur R. Menstrual hygiene, management, and waste disposal: practices and challenges faced by girls/women of developing countries. Journal of environmental and public health. 2018 Feb 20;2018
59	**155**	Newton J, Barnard G, Collins W. A rapid method for measuring menstrual blood loss using automatic extraction. Contraception. 1977 Sep 1;16(3):269–82
60	**150**	Hillard PJ. Menstruation in young girls: a clinical perspective. Obstetrics & Gynecology. 2002 Apr 1;99(4):655–62
61	**149**	Beausang CC, Razor AG. Young Western women’s experiences of menarche and menstruation. Health Care for Women International. 2000 Sep 1;21(6):517–28
62	**149**	Upashe SP, Tekelab T, Mekonnen J. Assessment of knowledge and practice of menstrual hygiene among high school girls in Western Ethiopia. BMC women's health. 2015 Dec;15:1–8
63	**147**	Koff E, Rierdan J. Preparing girls for menstruation: recommendations from adolescent girls. Adolescence. 1995 Dec 22;30(120):795–812
64	**147**	Hennegan J, Shannon AK, Rubli J, Schwab KJ, Melendez-Torres GJ. Women’s and girls’ experiences of menstruation in low-and middle-income countries: a systematic review and qualitative metasynthesis. PLoS medicine. 2019 May 16;16(5):e1002803
65	**147**	Singh AJ. Place of menstruation in the reproductive lives of women of rural North India. Indian Journal of Community Medicine. 2006 Jan 1;31(1):10–4
66	**146**	Omidvar S, Begum K. Factors influencing hygienic practices during menses among girls from south India: a cross sectional study. International Journal of Collaborative Research on Internal Medicine & Public Health. 2010;2(12):411–23
67	**145**	Garg S, Anand T. Menstruation related myths in India: strategies for combating it. Journal of family medicine and primary care. 2015 Apr;4(2):184
68	**144**	Sommer M. Ideologies of sexuality, menstruation and risk: girls’ experiences of puberty and schooling in northern Tanzania. Culture, health & sexuality. 2009 May 1;11(4):383–98
69	**143**	Ray S, Dasgupta A. Determinants of menstrual hygiene among adolescent girls: a multivariate analysis. National Journal of Community Medicine. 2012 Jun 30;3(02):294–301
70	**142**	Magalhaes J, Aldrighi JM, de Lima GR. Uterine volume and menstrual patterns in users of the levonorgestrel-releasing intrauterine system with idiopathic menorrhagia or menorrhagia due to leiomyomas. Contraception. 2007 Mar 1;75(3):193–8
71	**142**	Fraser IS, Warner P, Marantos PA. Estimating menstrual blood loss in women with normal and excessive menstrual fluid volume. Obstetrics & Gynecology. 2001 Nov 1;98(5):806–14
72	**141**	Aniebue UU, Aniebue PN, Nwankwo TO. The impact of pre-menarcheal training on menstrual practices and hygiene of Nigerian school girls. Pan African Medical Journal. 2009;2(1)
73	**140**	Kaunitz AM. Menstruation: choosing whether… and when. Contraception. 2000 Dec 1;62(6):277–84
74	**139**	Sommer M, Ackatia-Armah N, Connolly S, Smiles D. A comparison of the menstruation and education experiences of girls in Tanzania, Ghana, Cambodia and Ethiopia. Compare: A Journal of Comparative and International Education. 2015 Jul 4;45(4):589–609
75	**138**	Grant M, Lloyd C, Mensch B. Menstruation and school absenteeism: evidence from rural Malawi. Comparative education review. 2013 May 1;57(2):260–84
76	**138**	Burrows 1 A, Johnson S. Girls’ experiences of menarche and menstruation. Journal of Reproductive and infant Psychology. 2005 Aug 1;23(3):235–49
77	**138**	Merskin D. Adolescence, advertising, and the ideology of menstruation. Sex Roles. 1999 Jun;40(11–12):941–57
78	**132**	Haque SE, Rahman M, Itsuko K, Mutahara M, Sakisaka K. The effect of a school-based educational intervention on menstrual health: an intervention study among adolescent girls in Bangladesh. BMJ open. 2014 Jul 1;4(7):e004607
79	**130**	Stubbs ML. Cultural perceptions and practices around menarche and adolescent menstruation in the United States. Annals of the New York Academy of Sciences. 2008 Jun;1135(1):58–66
80	**128**	Nair P, Grover V, Kannan A. Awareness and practices of menstruation and pubertal changes amongst unmarried female adolescents in a rural area of East Delhi. Indian journal of community medicine. 2007 Apr 1;32(2):156-
81	**126**	Bharadwaj S, Patkar A. Menstrual hygiene and management in developing countries: taking stock. Junction Social. 2004 Nov 1;3(6):1–20
82	**125**	Menstrual hygiene and management in developing countries: taking stock: IRC. [cited 2023 Jun 21]. https://www.ircwash.org/resources/menstrual-hygiene-and-management-developing-countries-taking-stock
83	**123**	Miiro G, Rutakumwa R, Nakiyingi-Miiro J, Nakuya K, Musoke S, Namakula J, Francis S, Torondel B, Gibson LJ, Ross DA, Weiss HA. Menstrual health and school absenteeism among adolescent girls in Uganda (MENISCUS): a feasibility study. BMC women's health. 2018 Dec;18:1–3
84	**123**	Alam MU, Luby SP, Halder AK, Islam K, Opel A, Shoab AK, Ghosh PK, Rahman M, Mahon T, Unicomb L. Menstrual hygiene management among Bangladeshi adolescent schoolgirls and risk factors affecting school absence: results from a cross-sectional survey. BMJ open. 2017 Jul 1;7(7):e015508
85	**122**	Crofts T, Fisher J. Menstrual hygiene in Ugandan schools: an investigation of low-cost sanitary pads. Journal of water, sanitation and hygiene for development. 2012 Mar;2(1):50–8
86	**122**	Abioye-Kuteyi EA. Menstrual knowledge and practices amongst secondary school girls in lle lfe, Nigeria. The journal of the Royal Society for the Promotion of Health. 2000 Mar;120(1):23–6
87	**122**	Fraser IS, McCarron G, Markham R, Resta T. Blood and total fluid content of menstrual discharge. Obstetrics & Gynecology. 1985 Feb 1;65(2):194–8
88	**121**	Sharma A, Taneja DK, Sharma P, Saha R. Problems related to menstruation and their effect on daily routine of students of a medical college in Delhi, India. Asia Pacific Journal of Public Health. 2008 Jul;20(3):234–41
89	**114**	Zakherah MS, Sayed GH, El-Nashar SA, Shaaban MM. Pictorial blood loss assessment chart in the evaluation of heavy menstrual bleeding: diagnostic accuracy compared to alkaline hematin. Gynecologic and obstetric investigation. 2011;71(4):281–4
90	**111**	Bahram A, Hamid B, Zohre T. Prevalence of bacterial vaginosis and impact of genital hygiene practices in non-pregnant women in Zanjan, Iran. Oman medical journal. 2009 Oct;24(4):288
91	**108**	Phillips-Howard PA, Nyothach E, Ter Kuile FO, Omoto J, Wang D, Zeh C, Onyango C, Mason L, Alexander KT, Odhiambo FO, Eleveld A. Menstrual cups and sanitary pads to reduce school attrition, and sexually transmitted and reproductive tract infections: a cluster randomised controlled feasibility study in rural Western Kenya. BMJ open. 2016 Nov 1;6(11):e013229
92	**107**	R. Simes, DH Berg M. Surreptitious learning: menarche and menstrual product advertisements. Health care for women international. 2001 Jul 1;22(5):455–69
93	**105**	Bobhate PS, Shrivastava SR. A cross sectional study of knowledge and practices about reproductive health among female adolescents in an urban slum of Mumbai
94	**105**	Koff E, Rierdan J. Early adolescent girls' understanding of menstruation. Women & health. 1995 Jun 13;22(4):1–9
95	**104**	Demir SC, Kadayýfçý TO, Vardar MA, Atay Y. Dysfunctional uterine bleeding and other menstrual problems of secondary school students in Adana, Turkey. Journal of Pediatric and Adolescent Gynecology. 2000 Nov 1;13(4):171–5
96	**102**	Shah SP, Nair R, Shah PP, Modi DK, Desai SA, Desai L. Improving quality of life with new menstrual hygiene practices among adolescent tribal girls in rural Gujarat, India. Reproductive Health Matters. 2013 Jan 1;21(41):205–13
97	**101**	Juyal R, Kandpal SD, Semwal J, Negi KS. Practices of menstrual hygiene among adolescent girls in a district of Uttarakhand. Indian journal of community health. 2012 Jun 30;24(2):124–8
98	**100**	Phillips-Howard PA, Caruso B, Torondel B, Zulaika G, Sahin M, Sommer M. Menstrual hygiene management among adolescent schoolgirls in low-and middle-income countries: research priorities. Global health action. 2016 Dec 1;9(1):33032
99	**100**	Sapkota D, Sharma D, Pokharel HP, Budhathoki SS, Khanal VK. Knowledge and practices regarding menstruation among school going adolescents of rural Nepal. Journal of Kathmandu medical college. 2013;2(3):122–8
100	**100**	Higham JM, O'brien PM, Shaw R. Assessment of menstrual blood loss using a pictorial chart. BJOG: An International Journal of Obstetrics & Gynaecology. 1990 Aug;97(8):734–9

Bold values indicate the number of citations

## Abbreviations

MHH: Menstrual health and hygiene
LMICs: Lower-middle-income countries
MHM: Menstrual hygiene management
WASH: Water, Sanitation, and, Hygiene
